# Antibiotic Abuse and Antimicrobial Resistance in Hospital Environment: A Retrospective Observational Comparative Study

**DOI:** 10.3390/medicina58091257

**Published:** 2022-09-11

**Authors:** Patrizia Nardulli, Gabriel Gustafsson Hall, Alessandro Quarta, Giovanni Fruscio, Mariarita Laforgia, Vito M. Garrisi, Roberta Ruggiero, Salvatore Scacco, Danila De Vito

**Affiliations:** 1 IRCCS Istituto Tumori “G. Paolo II”, 70124 Bari, Italy; 2 Visby Hospital, Section of Clinical Microbiology and Infectious Diseases, Department of Medical Sciences, 62156 Visby, Sweden; 3 DLV System s.r.l., Research Section, Viale della Resistenza, 19, 87036 Quattromiglia, Italy; 4 Energent s.p.a., Research Section, Via Cristoforo Colombo, 112, 00154 Roma, Italy; 5 Tecnologica s.r.l., Research Section, 88900 Crotone, Italy; 6 Department of Basic Medical Sciences, Neurosciences and Sense Organs, University of Bari “Aldo Moro”, 70100 Bari, Italy; 7 School of Medicine, University of Bari “Aldo Moro”, 70100 Bari, Italy

**Keywords:** antimicrobial resistance, multidrug resistance, antibiotics, infections, bacterial isolates, clinical setting

## Abstract

*Background and Objectives*: Antimicrobial resistance represents a serious problem, and it may be life-threatening in the case of severe hospital-acquired infections (HAI). Antibiotic abuse and multidrug resistance (MDR) have significantly increased this burden in the last decades. The aim of this study was to investigate the distribution and susceptibility rates of five selected bacterial species (*E. coli*, *K. pneumoniae*, *P. aeruginosa*, *S. aureus* and *E. faecium*) in two healthcare settings located in the Apulia region (Italy). *Materials and Methods*: Setting n.1 was a university hospital and setting n.2 was a research institute working on oncological patients. All the enrolled patients were diagnosed for bacterial HAI. The observation period was between August and September 2021. Clinical samples were obtained from several biological sources, in different hospital wards. Bacterial identification and susceptibility were tested by using the software VITEC 2 Single system. *Results*: In this study, a higher incidence of multi-drug-resistant *K. pneumoniae* was reported (42,2% in setting n.1 and 50% in setting n.2), with respect to the Italian 2019 statistics report (30.3%). All the isolates of *E. faecium* and *S. aureus* were susceptible to linezolid. All the bacterial isolates of *P. aeruginosa* and most of *K. pneumoniae* were susceptible to ceftazidime–avibactam. Amikacin and nitrofurantoin represented a good option for treating *E. coli* infections. Multidrug-resistant (MDR) *P. aeruginosa*, methicillin-resistant *S. aureus* (MRSA) and *vancomycin-resistant* *E. faecium* (VRE) had a lower incidence in the clinical setting, with respect to *E. coli* and *K. pneumoniae. Conclusions*: The data obtained in this study can support clinicians towards a rational and safe use of antibiotics for treating the infections caused by these resistant strains, to enhance the overall efficacy of the current antibiotic protocols used in the main healthcare environments.

## 1. Introduction

Antimicrobial resistance is considered a worldwide impacting burden, affecting the patients of critical hospital wards, such as Intensive Care Units (ICU). Hospitalized patients have been demonstrated to have an increased risk to develop infections due to exposure to several invasive devices (mechanical ventilation, urinary tract catheters) [1,2,3,4] and to other related conditions. Careful clinical surveillance, together with the monitoring of the well-known bacterial strains responsible for inducing HAI, may help clinicians to choose the appropriate antibiotic therapies. The aim of this study was to investigate the distribution and susceptibility rates of five selected bacterial species in two healthcare settings located in the Apulia region (Southern Italy).

Bacterial infections have impacted humans throughout the centuries, until the discovery of antibiotics, which have revolutionized the treatment of infectious diseases. Because of their ability to survive in different environments, bacteria can increasingly face antibacterial treatments over time by means of different adaptative strategies [5,6,7,8,9]. They are able to modify the quaternary structure of specific target proteins, to substitute a metabolic pathway by synthesizing alternative biomolecules and to produce enzymes able to inactivate antibiotics; this is also possible through the camouflage of their structure, for example, behind a proteoglycan capsule [10,11,12,13].

A common bacterial weapon against penicillin is the beta-lactamase enzyme, which alters the beta-lactamic structure, thus maintaining the building of the bacterial wall and creating the local conditions to promote several diseases [14,15,16,17,18]. Bacteria are also able to synthesize effective isoforms of the beta-lactamase enzyme; the extended spectrum beta lactamase (ESBL) and the ESBL carbapenemase give bacteria resistance towards third-generation cephalosporins and carbapenamase-class antibiotics, respectively. These antibiotics are widely used in several nosocomial infections [19,20].

The spread of antibiotic-resistant bacterial strains is a severe issue for the healthcare systems of several countries [21,22]; it can be considered as an effect of antibiotics abuse [23,24,25]. Antibiotic-resistant bacteria are able to rapidly disseminate within the human body by transferring a ring of DNA to other species or strains [26,27,28].

According to the European Center for Disease Prevention and Control (ECDC), the most common and clinically relevant bacterial species in European hospitals include *E. coli*, *P. aeruginosa*, *K. pneumoniae*, *S. aureus* and *E. faecium.*

Common nosocomial infections involve the soft tissues, the urinary tract, the gastrointestinal organs and the respiratory apparatus. It is interesting to highlight that about 33% of patients receive antibiotics during their stay in hospital and about 6% undergo a hospital-acquired infection (ECDC) [29]. The spread of antibiotic resistance in Intensive Care Units (ICU), mainly due to the use of broad-spectrum antibiotics, has become a particular problem for clinicians and patients [29,30].

Among hospital-acquired infections in Europe, 41% of *S. aureus* infections are methicillin-resistant (MRSA), 24% of *E. coli* infections are resistant to cephalosporins (ESBL), 18% of *E. faecium* infections are resistant to vancomycin (VRE) and 32% of *P. aeruginosa* infections are resistant to carbapenems (ESBL-carba). Multidrug resistance (MDR) for *P. aeruginosa* is defined as a resistance to three different classes of antibiotics: beta-lactams (penicillin–tazobactam, cephalosporins or carbapenems), aminoglycosides and fluoroquinolones.

## 2. Materials and Methods

In this study, the antibiotic resistance of two hospital settings was compared.

Setting n.1 is a big University hospital, A.O.U.C. Policlinico in Bari, Southern Italy.

Setting n.2 is a Cancer Research Institute, IRCCS Istituto Tumori “Giovanni Paolo II” located in Bari, Southern Italy.

Both of the settings are located in the macro-region Apulia, Italy.

Based on the findings of ECDC, five of the most common pathogens in clinical settings have been chosen to build up this survey: *E. coli*, *K. pneumoniae*, *P. aeruginosa*, *S. aureus* and *E. faecium*. The inclusion criteria have been described below.

Within setting n.1, clinical samples were obtained by four different departments (Neonatology, Infectious Disease, Intensive Care Unit and Internal Medicine). Within setting n.2, clinical samples were obtained by all departments (surgery, oncology, hematology, interventistic oncology).

The most suitable clinical samples and materials to correlate with a potential clinical infection were blood, urine and respiratory cultures, rectal swabs and samples extracted from devices implanted into the patient’s body, such as venous catheters. All patients were included, regardless of age, but multiple samples of the same date for the same patient with the same result were considered only once in the report.

From 30 August to 30 September 2021 (only one month, to easily build a research framework), clinical samples were collected retrospectively from the software VITEC 2 Single system (BioMérieux, Inc, Hazelwood, Mo, USA). The bacteria were identified by using VITEC MS (MALDI system, BioMérieux, Grassina, Italy). MALDI-TOF-MS uses the software Mass-Up, distributed under license GPLv3 [31]. The antibiograms were achieved starting from standard dilution in physiological solution to 0.53–0.67 density; then, the samples were cultured overnight, with antibiogram cards for determination of Minimal inhibitory concentration (MIC) values and interpreted to Eucast European Committee On Antimicrobial Susceptibility Testing (EUCAST) clinical breakpoints for susceptibilty. ESBL phenotype was defined as resistance to cefotaxime or ceftazidime and inhibition by ESBL inhibitor such as tazobactam. Finally, we have demonstrated the MIC results of bacterial strains examined by using ETEST (BioMérieux, Italy) [31].

## 3. Results

All data are reported in Table 1 for setting n.1 and Table 2 for setting n.2.

### 3.1. E. coli

In setting n.1, during the reported timelapse, 43 different bacterial colonies of *E. coli* were found, and 30.2% (13/43) of them were ESBL-producing, showing a cefotaxime or ceftazidime resistance and a susceptibility to ESBL inhibitors. The isolated bacteria were mainly derived from urine cultures (67.4%—29/43) and blood cultures (18.6%—8/43). Several samples were obtained from Internal Medicine (55.8%—24/43). The rate of resistance was ampicillin 66.6% (26/39), amoxicillin–clavulanic acid 46.5% (20/43) and fluoroquinolone 41.8% (18/43). Carbapenem resistance was rare (2%—1/43). All the isolated bacteria were sensitive to nitrofurantoin (37/37) and ertapenem (34/34). The resistance rate to gentamycin (18.6%—8/43) was higher than amikacin (4.6%—2/43).

In setting n.2, among the six isolated bacteria, only two (33.3%), deriving both from urine samples, showed antibiotic resistance to cephalosporins and penicillin in the oncology department, showing no other resistance patterns.

### 3.2. E. faecium

In setting n.1, only 10 samples reporting *E. faecium* were recorded, among which 30% (3/10) were vancomycin-resistant (VRE). The most common material was obtained from venous catheter cultures (40%—4/10). All strains were resistant to amoxicillin–clavulanic acid and imipenem (10/10). Fluoroquinolone resistance was common 70% (7/10), while all bacterial isolates were sensitive to linezolid (10/10), followed by tigecycline (9/10) and teicoplanin (8/10).

No cases of resistant *E. faecium* strains were observed in setting n.2.

### 3.3. S. aureus

In setting n.1, 20 bacteria of *S. aureus* were isolated; among these, 40% (8/20) were methicillin-resistant (MRSA). The most common source material was wound cultures (40%—8/20) and came from Internal Medicine wards (60%—12/20).

The rate of resistance was penicillin 65% (13/20), clindamycin 50% (10/20), erythromycin 55% (11/20), tetracycline 20% (4/20) and rifampicin 10% (2/20). No resistance to trimethoprim-sulfamethoxazole was observed, but one case of increased exposure was reported.

All bacterial isolates were susceptible to linezolid, teicoplanin, daptomycin, vancomycin and tigecycline.

No cases of *S. aureus* resistance were observed in setting n.2.

### 3.4. Pseudomonas aeruginosa

In setting n.1, the number of *P. aeruginosa* infections was 27; among these, 11% (3/27) were multidrug-resistant (MDR). The most common source material was respiratory samples (29.6%—8/27). The most isolates came from Internal Medicine wards (44%—12/27) and ICU (33.3%—9/27), with respiratory samples as the most common source (44%—4/9).

Among the isolated bacteria, 25.9% (7/27) were ceftazidime-resistant, 22.2% (6/27) were piperacillin–tazobactam-resistant, 18.5% (5/27) were ciprofloxacin-resistant and 25.9% (7/27) were carbapenem-resistant strains.

Aminoglycoside resistance was rare (3.7–7.4%—1/27 and 2/27 for amikacin and tobramycin, respectively), and all samples were sensitive to ceftazidime–avibactam and colistin (27/27).

No cases of resistant *P. aeruginosa* strains were observed in setting n.2.

### 3.5. K. pneumoniae

In setting n.1, the most numerous bacterial infections in the considered period were induced by *K. pneumoniae*, with 45 clinical isolates. Among them, 42.2% (19/45) were multidrug-resistant (MDR). The most common source was urine cultures (46.6%—21/45), especially from Internal Medicine wards (12/21). The samples came from Internal Medicine wards (18/45) and ICU (18/45). From ICU, respiratory samples were the most common source (33.3%—6/18).

As for antibiotic resistance, 53.3% (24/45) were resistant to ceftazidime, 44.4% (20/45) to fluoroquinolones and trimethoprim–sulfamethoxazole, 42.2% to carbapenems and 35.5–37.7% to aminoglycosides (16/45 for gentamycin and 17/45 for amikacin, respectively). Moreover, 26.6% of samples (12/45) were resistant to ceftazidime–avibactam and 13.3% (6/45) were resistant to colistin.

Data from setting n.2 showed four bacterial isolates of MDR *K. pneumoniae* infections from a blood culture and three rectal swabs, with an incidence rate of 50% (4/8). *K. pneumoniae* cases overlap with data from setting n.1 in terms of multidrug resistance to antibiotics; in particular, all strains were resistant to cephalosporins, fluoroquinolones, carbapenems and aminoglycosides.

## 4. Discussion

The results of the surveillance on antimicrobial resistance obtained in this study conducted in Apulia are in agreement with the 2019 national report. According to ECDC, the proportion of resistant bacterial isolates of each species was higher than the European average for hospital-acquired infections, except for MRSA, which was similar (40–41%) [12,13,14,15,16].

The different trend principally refers to the higher incidence of multidrug-resistant *K. pneumoniae*, recognized by clinicians as a very difficult challenge [32]. In detail, the Italian 2019 statistics report that 30.3% of *K. pneumoniae* was MDR, while in this study the rate was closer to 50% both in setting n.1 (42.2%) and in setting n.2 (50%). In the report study conducted in setting n.2, it is evident there is a correlation between MDR *K. pneumoniae* infections and low immunity defense in hematologic patients [33,34]. In setting n.1, extensively resistant strains of *K. pneumoniae*, susceptible to only one or two antibiotics such as colistin or amikacin, were not rare. The combination of MDR *K. pneumoniae* with the few treatment options and its prevalence in respiratory and urine cultures still represent a great safety problem for clinicians [15,16,17,18].

Within the data collected, the species here investigated were shown to promote infections in different biological sites: *E. faecium* was prevalently isolated from venous catheter cultures, *P. aeruginosa* from respiratory cultures, *S. aureus* from wound cultures and *K. pneumoniae* and *E. coli* were mainly isolated from urine cultures (and rectal swabs for setting n.2) [7,12,19,22].

In setting n.1, Internal Medicine wards produced more cultures, probably due to the number of patients. Predomination of *K. pneumoniae* and *P. aeruginosa* was evident in ICU and in respiratory cultures with respect to other species. The cultures from Neonatology included a larger number of *S. aureus* and *E. coli* infections.

All isolates of *E. faecium* and *S. aureus* were susceptible to linezolid, which could be an alternative to vancomycin as empirical treatment for resistant Gram-positive pathogens (30% of VRE). *S. aureus* strains were also sensitive to teicoplanin, tigecycline, vancomycin and daptomycin. No resistant strains of *S. aureus* to trimethoprim–sulfamethoxazole were observed.

All isolates of *P. aeruginosa* and most of *K. pneumoniae* were susceptible to ceftazidime–avibactam. Therefore, this association represents a potent weapon in the treatment of resistant Gram-negative infections. *E. coli* strains were not tested for ceftazidime–avibactam, having many other treatment options, including carbapenems [33,34]. 

The results also indicate that amikacin could be a good choice for treating *E. coli* infections, with only 4.6% resistance compared to 18.6% gentamycin resistance. 

Nitrofurantoin remains an excellent treatment option for uncomplicated cystitis caused by *E. coli*, even in hospital settings; in fact, no resistant strains were observed in this study.

## 5. Conclusions

In this study, patients with MDR bacterial infections were selected across various hospital wards. Samples collected from these medical departments gave us a panorama of which kinds of bacterial strains must undergo continuous surveillance. Oncological and hematological, Intensive Care Unit and Neonatology patients have an especially high risk of infections with bacterial resistance to most common antibiotics. In these patients, the most proper empirical antibiotic therapy must be applied with the aim to enhance efficacy and decrease forms of resistance. Data obtained from the current studies can address the rational use of antibiotics by clinicians by avoiding the inappropriate use of non-active antibiotics.

In conclusion, compared to *E. coli* and *K. pneumoniae*, MDR *P. aeruginosa*, MRSA *S. aureus* and VRE *E. faecium* certainly present a lower incidence in the clinical setting (no cases in setting n.2). The effective management of these resistant strains and a correct antibiotic therapy based on the resistance’s epidemiology represent the most potent clinical approach to enhance the efficiency of antibiotics and to reduce bacteria-associated mortality.

### Future Insights

Healthcare-associated infections (HAIs) are one of the most impacting causes of preventable death and disability within the hospitalized population.

Recently, several strategies have been proposed to face this challenge, such as strong prevention or the support of computer-based analyses. The EU’s call for projects has also promoted the development of innovative artificial intelligence (AI) solutions to prevent infections inside clinical departments. In particular, an interesting ongoing project (LAOCOONTE project, by Energent S.p.A.) has the objective to develop specific use cases, where data can be used by machine and deep learning models to evaluate the likelihood of infection in clinical departments, in an Italian clinical setting. This approach is really promising; in fact, nowadays, AI acts an important role in different fields from smart manufacturing to the Internet of things, human–computer interaction and medical scenarios, of course. The attention of the scientific community and industry to the AI field is related to the excellent performance achieved in recent years by the so-called artificial neural networks, in particular the deep architectures, in various fields such as text, images and audio [31,32,33]. Nosocomial infections (NIs) are even more preventable, as they represent a biological and social cost for hospitalized patients. The growing availability of computerized patient records in hospitals allows for the improvement of data storage with traditional machine learning methods, which have been shown to outperform deep learning’s performance when applied to tabular data. 

The objective of the future is to understand how to prevent the causes underlying NIs and to increase safety procedures once patients have been admitted to hospitals.

## Figures and Tables

**Table 1:** Description of bacterial isolates per species including departments, source material and resistance patterns against selected antibiotics in setting n.1 (30 August–30 September 2021).

Species	*S. aureus*	*E. faecium*	*K. pneumoniae*	*P. aeruginosa*	*E. coli*
Number of bacterial isolates Total *n* = 145	20	10	45	27	43
Department					
Medicine	60% (12/20)	60% (6/10)	40% (18/45)	44% (12/27)	55.8% (24/43)
Intensive care unit	10% (2/20)	20% (2/10)	40% (18/45)	33.3% (9/27)	11.6% (5/43)
Infectious disease	15% (3/20)	20% (2/10)	15.5% (7/45)	18.5% (5/27)	23.2% (10/43)
Neonatology	15% (3/20)	0%	4.4% (2/45)	3.7% (1/27)	9.3% (4/43)
Source material					
Urine	5% (1/20)	20% (2/10)	46.6% (21/45)	14.8% (4/27)	67.4% (29/43)
Blood	30% (6/20)	30% (3/10)	11% (5/45)	22.2% (6/27)	18.6% (8/43)
Respiratory	25% (5/20)	0%	22.2% (10/45)	29.6% (8/27)	2.3% (1/43)
Wound	40% (8/20)	10% (1/10)	13.3% (6/45)	18.5% (5/27)	4.6% (2.43)
Venous catheter	0%	40% (4/10)	6.7% (3/45)	7.4% (2/27)	7.0% (3/43)
Resistance to antibiotics					
	40% (8/20) methicillin-resistant *S. aureus* (MRSA)	30% (3/10) *vancomycin-resistant* *E. faecium* (VRE)	42.2% (19/45) multidrug resistance (MDR)	11% (3/27) MDR	30.2% (13/43) extended spectrum beta lactamase (ESBL)
Benzylpenicillin	65% (13/20)				
Ampicillin					66.6% (26/39)
Amoxicillin–Clavulanic Acid		100% (10/10)	60% (27/45)		46.5% (20/43)
Piperacillin–tazobactam			55.5% (25/45)	22.2% (6/27)	7.0% (3/43)
Cefotaxime			51.1% (23/45)		30.2% (13/43)
Ceftazidime			53.3% (24/45)	25.9% (7/27)	18.6% (8/43)
Carbapenems (imipenem or meropenem)		100% (10/10)	42.2% (19/45)	25.9% (7/27)	2% (1/43)
Ceftazidime–avibactam			26.6% (12/45)	0%	
Clindamycin	50% (10/20)				
Amikacin			37.7% (17/45)		4.6% (2/43)
Gentamycin	20% (4/20)		35.5% (16/45)		18.6% (8/43)
Fluoroquinolones (ciprofloxacin or levofloxacin)	30% (6/20)	70% (7/10)	44.4% (20/45)	18.5% (5/27)	41.8% (18/43)
Linezolid	0%	0%			
Vancomycin	0%	30% (3/10)			
Daptomycin	0%	0%			
Tigecycline	0%	10% (1/10)			
Tetracycline	20% (4/20)				
Trimethoprim–sulfamethoxazole	0%		44.4% (20/45)		
Nitrofurantoin					0%
Colistin			13.3% (6/45)	0%	

**Table 2:** Description of bacterial isolates per species, source material and resistance patterns against selected antibiotics in setting n.2 (30 August–30 September 2021).

Species	*K. pneumoniae*	*E. coli*
Number of bacterial isolates Total *n* = 14	8	6
Department		
Hematology	100% (8/8)	0%
Interventional oncology	0%	100% (6/6)
Source material		
Urine	0%	100% (6/6)
Blood	25% (2/8)	0%
Rectal swab	75% (6/8)	0%
Resistance to antibiotics		
	50% (4/8) MDR	33.3% (2/6) ESBL
Ampicillin		33.3% (2/6)
Amoxicillin–clavulanic Acid	50% (4/8)	33.3% (2/6)
Piperacillin–tazobactam	50% (4/8)	33.3% (2/6)
Cefotaxime	50% (4/8)	33.3% (2/6)
Ceftazidime	50% (4/8)	33.3% (2/6)
Carbapenems (imipenem or meropenem)	50% (4/8)	0%
Ceftazidime–avibactam	50% (4/8)	
Amikacin	50% (4/8)	0%
Gentamycin	50% (4/8)	0%
Fluoroquinolones (ciprofloxacin or levofloxacin)	50% (4/8)	0%
Trimethoprim–sulfamethoxazole	50% (4/8)	
Nitrofurantoin		0%
Colistin	0%	

## Data Availability

Each author received a personal copy of the Eclipse IDE, equipped with the Log Processor plugin, and personal access to the GitHub platform. The team of each setting created a personal private repository on GitHub where they have uploaded any data. The comprehensive datasets are available in the private GitHub platform, stored by D.D.V.
